# Best practice guidelines for idiopathic nephrotic syndrome: recommendations versus reality

**DOI:** 10.1007/s00467-014-2903-7

**Published:** 2014-08-17

**Authors:** Andrea Pasini, Gabriella Aceto, Anita Ammenti, Gianluigi Ardissino, Vitalba Azzolina, Alberto Bettinelli, Elena Cama, Sante Cantatore, Antonella Crisafi, Giovanni Conti, Maria D’Agostino, Alessandra Dozza, Alberto Edefonti, Carmelo Fede, Elena Groppali, Chiara Gualeni, Alessandra Lavacchini, Marta Lepore, Silvio Maringhini, Paola Mariotti, Marco Materassi, Francesca Mencarelli, Giovanni Messina, Amata Negri, Marina Piepoli, Fiammetta Ravaglia, Angela Simoni, Laura Spagnoletta, Giovanni Montini, On behalf of the NefroKid Study Group

**Affiliations:** 1 Nephrology and Dialysis Unit, Department of Pediatrics, Azienda Ospedaliero Universitaria Sant’Orsola-Malpighi, Via Massarenti 11, 40138 Bologna, Italy; 2 Nephrology Unit, Giovanni XXIII Children’s Hospital, Bari, Italy; 3 Department of Pediatrics, University of Parma, Parma, Italy; 4 Pediatric Nephrology and Dialysis Unit, Fondazione Ca’ Granda IRCCS Ospedale Maggiore Policlinico Milano, Milan, Italy; 5 Pediatric Nephrology Unit, Children’s Hospital ‘G. Di Cristina’, A.R.N.A.S. ‘Civico’, Palermo, Italy; 6 Pediatric Unit, San Leopoldo Mandic Hospital, Merate, Italy; 7 Department of Pediatrics and Neonatology, Desenzano del Garda, Italy; 8 Department of Pediatrics, Azienda Ospedaliera-University of Modena, Modena, Italy; 9 Pediatric Unit, Santa Maria Nuova Hospital, Reggio Emilia, Italy; 10 Pediatric Nephrology and Dialysis, Azienda Ospedaliera Universitaria Di Messina G. Martino, Messina, Italy; 11 Pediatric Unit, S. Giovanni XXIII Hospital, Bergamo, Italy; 12 Pediatric Unit, Ospedale Maggiore, Bologna, Italy; 13 Pediatric Unit, Children’s Hospital, Brescia, Italy; 14 Pediatric Unit, Ospedale degli Infermi, Rimini, Italy; 15 Pediatric Unit, San Jacopo Hospital, Pistoia, Italy; 16 Nephrology and Dialysis Unit, Meyer Children’s Hospital, Florence, Italy; 17 Pediatric Unit, Filippo Del Ponte Hospital, Varese, Italy; 18 Pediatric Unit, Guglielmo da Saliceto Hospital, Piacenza, Italy; 19 Pediatric Unit, Ramazzini Hospital, Carpi, Italy

**Keywords:** Idiopathic nephrotic syndrome, Steroid regimen, Albumin infusion, Thromboembolic prophylaxis, Guidelines

## Abstract

**Background:**

The optimal therapeutic regimen for managing childhood idiopathic nephrotic syndrome (INS) is still under debate. We have evaluated the choice of steroid regimen and of symptomatic treatment adopted by pediatricians and pediatric nephrologists in a large number of centers as the first step towards establishing a shared protocol

**Methods:**

This was a multicenter, retrospective study. A total of 231 children (132 admitted to pediatric units) aged 6 months to <15 years who presented with onset of nephrotic syndrome to 54 pediatric units and six pediatric nephrology units in Italy between 2007 and 2009 were eligible for entry into the study.

**Results:**

Median steroid dosing was 55 (range 27–75) mg/m^2^/day. The overall median cumulative dose regimen for the first episode was 3,440 (1,904–6,035) mg/m^2^, and the median duration of the therapeutic regimen was 21 (9–48) weeks. The total duration and cumulative steroid dose were significantly higher in patients treated by pediatricians than in those treated by pediatric nephrologists (*p* = 0.001 and *p* = 0.008). Among the patient cohort, 55, 64 and 22 % received albumin infusions, diuretics and acetyl salicylic acid treatment, respectively, but the laboratory and clinical data did not differ between children treated or not treated with symptomatic drugs. Albumin and diuretic use did not vary between patients in pediatric units and those in pediatric nephrology units.

**Conclusions:**

This study shows major differences in steroid and symptomatic treatment of nephrotic syndrome by pediatricians and pediatric nephrologists. As these differences can influence the efficacy of the treatments and the appearance of side-effects, shared guidelines and their implementation through widespread educational activities are necessary.

## Introduction

Idiopathic nephrotic syndrome (INS) is a rare disease (2–7 cases/year per 100,000 children aged <14 years), characterized by edema, massive proteinuria and hypoalbuminemia [1–3]. Its rarity makes the management of the disease cumbersome, especially in non-specialized centers. The current mainstream therapy consists of corticosteroids, which induce remission in 90–95 % of children; however, approximately 50 % of these frequently relapse and become steroid-dependent [4, 5]. The first therapeutic approach [4 weeks of daily prednisone (PDN), followed by 4 weeks of PDN on 3 consecutive days per 7 days) was proposed in 1966, and since then varying steroid regimens have been suggested, with the aim of influencing the course of the disease [6–10]. In 2000, a systematic review showed that the risk of relapse may be reduced by longer treatment duration and a higher cumulative steroid dose [5]. Various local, national and international clinical practice guidelines have been published based on currently available evidence [11–15], but in Italy, a shared protocol has not yet been established.

In the early phase of the disease, symptomatic treatment is also important as many severe complications can occur (infections, thromboembolism, hypovolemia), which are either directly related to the pathophysiology of the underlying NS or to the steroid treatment itself. To date, very few studies—most of which involve only a small number of patients—have been published on the prophylaxis and treatment of these early complications, while recommendations are either lacking or conflicting [16–23]. Moreover, most of the published data relates to cohorts of children treated at referral units.

For these reasons, the aim of this study was to retrospectively evaluate the different therapeutic strategies adopted by pediatricians and pediatric nephrologists in a large number of centers, as the first step towards establishing a shared protocol.

## Methods

Pediatricians from 114 pediatric units (PUs) and six pediatric nephrology Units (PNUs) in nine Italian regions (Lombardy, Emilia-Romagna, Tuscany, Apulia, Sicily, Marche, Trentino, Friuli, Sardinia) were invited to participate in the study, after obtaining approval from their respective Ethics Committees. The participating pediatricians were asked to add the clinical, epidemiological and laboratory data of their patients to an online database (www.nefrokid.it) in a pseudonymized manner.

The inclusion criteria were: (1) onset of INS between January 2007 and December 2009, with INS defined as proteinuria of >40 mg/m^2^/h or a urinary protein/creatinine ratio (PrU/CrU) of >2 mg/mg, hypoalbuminemia (<2.5 g/dL) and edema; (2) age at onset: ≥0.5 to < 15 years (thus excluding children with congenital forms of NS and including all late-onset INS.

Subjects diagnosed with secondary NS, the presence of low C3 and C4 levels, congenital NS or NS in association with syndromes were excluded. All of the PNUs had patients who met the inclusion criteria, whereas only 60/114 PUs had suitable patients.

At diagnosis, height, weight, body mass index (BMI), systolic (sBP) and diastolic blood pressure (dBP), expressed as standard deviation scores (SDS), and laboratory data (blood glucose, creatinine, urea, uric acid, total protein, albumin, alpha-2globulin, gamma-globulin, total cholesterol, high-density lipoprotein, low-density lipoprotein, triglycerides, Na, K, Ca, P, complete blood count, prothrombin time-partial thromboplastin time, fibrinogen, anti-thrombin III, platelets, microscopic hematuria, proteinuria, urinary creatinine and diuresis) were collected. Days of hospitalization, number of blood tests performed and blood tests/days of hospitalization were compared among participating centers and between PUs and PNUs.

### Epidemiological data

Morbidity rates were based on the data from the Emilia Romagna region, where all of the PUs and PNUs participated in the study. The incidence rate was calculated as the incidence per calendar year/100,000 age-related population (≥0.5 to <15 years). The given incidence refers to the data collected during the 3 years of the study (2007/2008/2009). All of the regional units were contacted and asked to search for patients registered with the code DRG 581-582-583 (for NS, chronic glomerulonephritis and chronic nephropathies). The same codes were then crosschecked against the regional database. The files of the patients identified were evaluated to confirm the diagnosis of INS. The estimated number of people “at risk” according to the morbidity analysis was derived from the 2009 Census as 533,890.

### Steroid regimens

The different steroid regimens utilized were evaluated.

Induction therapy was defined as the daily use (in single or divided doses) of PDN at the highest fixed dose (mg/m^2^). We looked for the implementation of the 4- and 6-week (±3 days) induction regimens, as recommended by the International Study of Kidney Disease in Children (ISKDC) [6] and the Arbeitsgemeinschaft für Pädiatrische Nephrologie (APN) [11], respectively.

Maintenance therapy was considered to be the use of a stable dose of PDN given on alternate days and/or a tapered dose.

The total length (induction and maintenance therapy) of the steroid regimen and total PDN dose (mg/m^2^) were evaluated. Data from PUs and PNUs were compared.

Remission was defined as the disappearance of proteinuria, based on daily urinary dipstick results reported as “negative” or “trace,” for at least 3 consecutive days. The time to remission was defined as the number of days between the first steroid dose and the first day with a “negative”–“trace” dipstick result. Relapse was defined as a dipstick result of 3+ for at least 3 consecutive days, or a PrU/CrU of >2.

### Symptomatic therapy

The number and dosage of albumin infusions, diuretics, antihypertensive drugs, calcium-containing medications, prophylactic anticoagulant therapy and vitamin D were evaluated and then correlated with clinical and laboratory parameters. Their possible influence on time to remission was evaluated, and the different ways in which pediatricians and pediatric nephrologists utilize symptomatic therapies were compared. Data from PUs and PNUs were compared.

### Statistical analysis

All data were analyzed using Stata9.2 (StataCorp, College Station, TX). Patient characteristics are presented as the mean ± standard deviation, median and range or percentage. Differences in proportions in the categorical data were tested using the chi-square test (Fisher test and Pearson test), while differences for continuous outcome measures were tested using Student’s *t* test or the Kruskal–Wallis test, as appropriate. The null hypothesis was rejected for all tests with two-tailed alpha values <0.05. The differences between treatments and the 95% confidence interval (CI) for this difference were calculated. Risk factors were reported as an odds ratio (OR) with 95 % CI.

## Results

The patient cohort comprised 231 children who both satisfied the inclusion criteria and received treatment at one of the six PNUs or 60/114 PUs which submitted data to the online database for further evaluation between January 2007 and December 2009. All of the patients in our study were hospitalized at onset, with 60 % admitted to the PU and 40 % to the PNU. The median duration of hospitalization was 10.7 (range 2–35) days, and there was a median of 0.5 (range 0.1–1.5) blood samples per day of hospitalization, with no differences between patients admitted to the PU and those admitted to the PNU. Epidemiological, clinical and laboratory characteristics at onset are shown in Table 1. The incidence rate (data from the Emilia Romagna Region) was 3.5/100,000 age-related population, and median age at diagnosis was 3.7 (0.7–14.9) years. The median time of response to PDN treatment was 10 (range 2–81) days.

**Table 1:** Epidemiological, clinical and laboratory parameters of pediatric patients at onset of idiopathic nephrotic syndrome

Characteristics of patient cohort	Number of patients for which specific data were available	Total patient cohort (*n* = 231)	Pediatric units (*n* = 135)^a^	Pediatric nephrology units (*n* = 96)^a^
Age at diagnosis (year)	231	4.7 ± 3.06	5.05 ± 3.1	4.2 ± 2.6
Sex (male:female)	231	157:74 (68 %:32 %)	88:47 (65 %:35 %)	69:27 (72 %:28 %)
Race	231			
Caucasian		196	106	90
Asian		6	5	1
African		16	12	4
Hispanic		7	7	0
Arabian		6	5	1
Season (autumn/winter vs. spring/summer	231	115:116	74:68	41:48
Clinical characteristics (SDS)					
Height	231	−0.08 ± 1.06	−0.01 ± 1.08	−0.2 ± 1
Weight	231	0.5 ± 1.05	0.56 ± 0,95	0.5 ± 1.2
BMI	231	0.74 ± 0.94	0.77 ± 0.94	0.7 ± 0.9
Systolic blood pressure	197	1.05 ± 1.1 8	1.17 ± 1.04	0.9 ± 1.3
Diastolic blood pressure	196	1.23 ± 0.9	1.26 ± 0.81	1.2 ± 1.1
Urine output (mL/kg/h)		1.4 ± 1.0	1.3 ± 0.9	1.7 ± 1.2
Laboratory data					
Urea (mg/dL)	212	29.6 ± 14	29.2 ± 14.2	30.3 ± 13.8
Creatinine (mg/dL)	212	0.35 ± 0.15	0.35 ± 0.15	0.4 ± 0.2
Uricemia (mg/dL)	88	4.2 ± 1.1	4.45 ± 1.0	4.0 ± 1.1
Total proteins (g/dL)	212	4.2 ± 0.7	4.2 ± 0.7	4.2 ± 0.7
Albumin (g/dL)	169	1.4 ± 0.4	1.4 ± 0.4	1.4 ± 0.4
Alpha-2- globulin (%)	112	29.8 ± 12.5	30.2 ± 12.8	29.8 ± 12.4
Gamma-globulin (%)	141	7.3 ± 2.3	7.2 ± 2.4	7.5 ± 2.1
Total cholesterol (mg/dL)	208	401 ± 100.3	414 ± 103.8	381.3 ± 92
Triglycerides (mg/dL)	183	211 ± 133	216 ± 144	205.5 ± 117.3
Na (mmol/L)	206	136 ± 3.2	136.1 ± 3.1	136.1 ± 3.6
K (mmol/L)	206	4.5 ± 0.5	4.5 ± 0.5	4.6 ± 0.6
Ca (mg/dL)	199	8.1 ± 0.7	8.1 ± 0.6	8.3 ± 0.8
P (mg/dL)	149	5 ± 1	4.9 ± 1	5.2 ± 1.1
Fibrinogen (mg/dL)	143	659 ± 1,240	702,4 ± 232,2	587.3 ± 236.7
Antithrombin III (activity %)	66	69.5 ± 24.2	63,4 ± 2.5	80 ± 25.6
Platelets (×10^3^/μL)	207	432 ± 131	412.2 ± 137.1	463.3 ± 116.8
PrU/CrU (mg/mg)	112	10.8 ± 7.55	10.5 ± 7.1	11.3 ± 8.2
Microhematuria (yes)	231	94 (41 %)	49 (36 %)	45 (47 %)

SDS, Standard deviation score; BMI, body mass index; PrU/CrU, urinary protein/creatinine ratio

Data in table are presented as the number of patients with the percentage in parenthesis, or as the mean ± standard deviation. All data refer to data collected at initial admission

^a^No differences were found between the two groups of patients [pediatric unit (PU) and pediatric nephrology unit (PNU)], apart from age at diagnosis (*p* = 0.042)

**Fig. 1:**
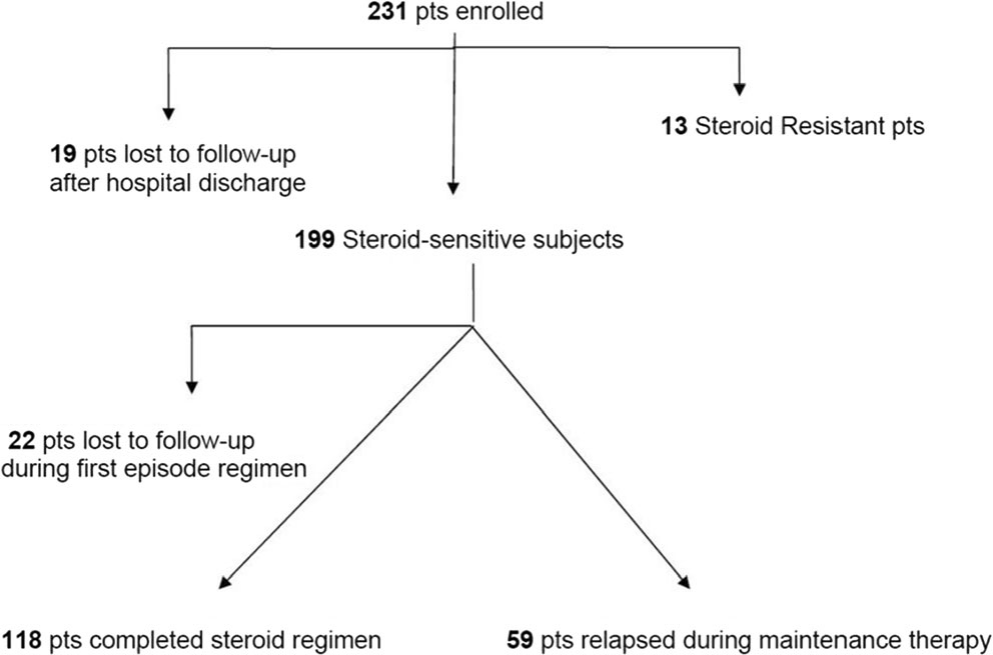
Flowchart of the 231 enrolled pediatric patients

The flowchart of the 231 enrolled patients for the duration of the study is shown in Fig. 1.

### Steroid treatment

**Fig. 2:**
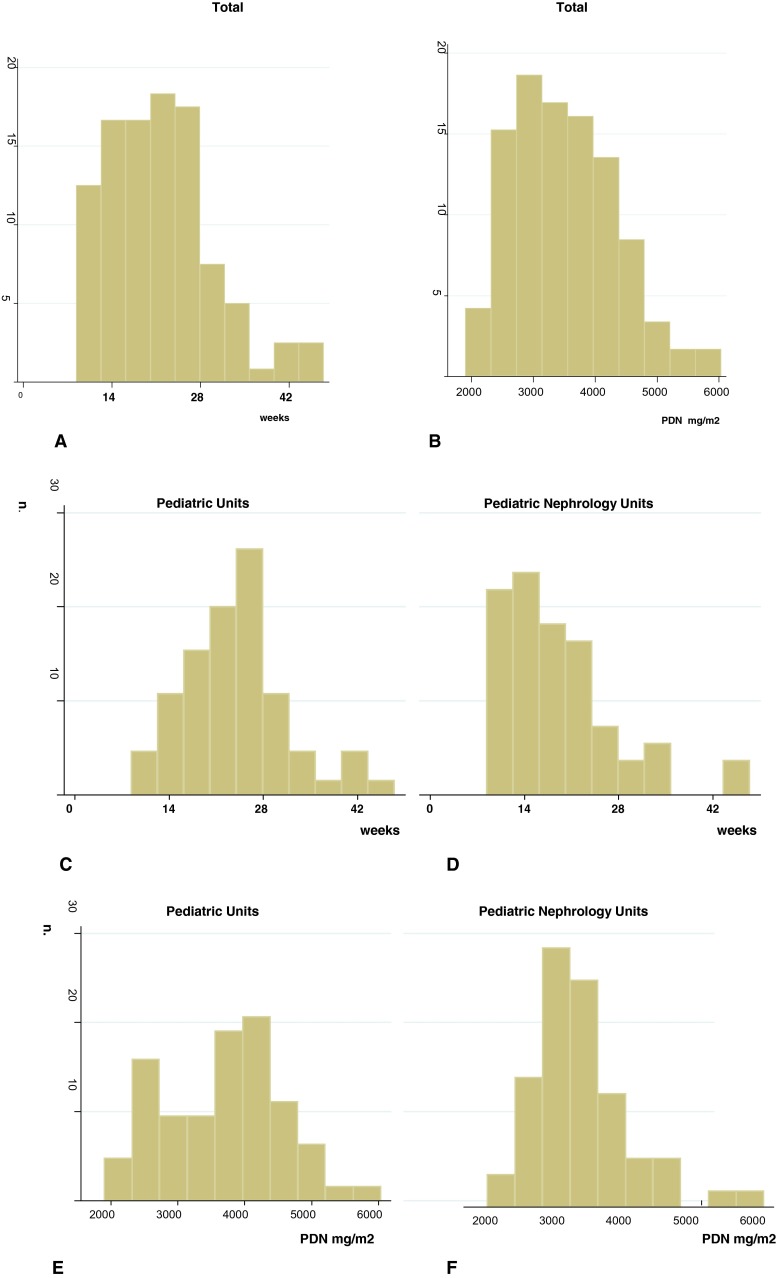
Treatment regimen for the first episode.** a**,** b** Duration (**a**) and total prednisone dose utilized (**b**) for the total patient cohort.** c**–**f** Comparison of the duration (**c**, **d**) and total prednisone dose (**e**, **f**) of the steroid regimen for the first episode used by participating pediatricians (**c**, **e**) and pediatric nephrologists (**d**, **f**).* Y-axis* shows the number of patients.* PDN* Prednisone

The steroid treatment regimens reported by the pediatricians at the different participating PUs and PNUs differed in terms of PDN dose and duration (Fig. 2; Table 2).

**Table 2:** Comparison of steroid regimens (induction, maintenance and total treatment) utilized by pediatricians and pediatric nephrologists

Variable	Steroid regimen	Children admitted to PU (*n* = 132)	Children admitted to PNU (*n* = 86)	*p* value
Weeks	Induction	5 (2.5–8)	6 (4–11)	0.0001
	Maintenance	20 (5–39.5)	14 (3–44)	0.0003
	Total	24 (9–45.5)	20 (10–48)	0.0014
PDN (mg/m^2^)	Induction	1,887 (756–4,260)	2,117 (1,092–5,341)	0.012
	Maintenance	1,883 (657–4,235)	1,259 (525–3,321)	0.0001
	Total	3,722 (1,904–6,035)	3,301 (2,250–5,660)	0.008

PDN, Prednisone; PU, pediatric unit; PNU, pediatric nephrology unit

Values are presented as the median with the range given in parenthesis

#### Induction treatment

Prednisone was administered at a median dose of 55.2 (range 27–75) mg/m^2^/day; however, cases of underdosing and overdosing (from 27 to 75 mg/m^2^) were noted. Comparison of the PDN dosing regimen used in the PU and PNU revealed a significant difference (60 vs. 53.5 mg/m^2^; *p* = 0.01). PDN was administered for a period of 4 (3–11) weeks with a cumulative dose of 1,800 (756–5,341) mg/m^2^. Of the 199 steroid-sensitive (SS) children, 93 (46.7 %) followed a 4-week induction regimen, 53 (26.6 %) a 6-week induction regimen and 53 (26.6 %) either a shorter or longer induction regimen.

#### Maintenance treatment

Median duration of the maintenance treatment was 16 (3–44) weeks, and the PDN cumulative dose was 1,410 (525–4,235) mg/m^2^.

#### Overall treatment

The overall median duration of the treatment regimen for the first episode was 21 (9–48) weeks (Fig. 2a), and the median cumulative PDN dose was 3,440 (1,904–6,035) mg/m^2^ (Fig. 2b). No patient was treated for less than 8 weeks, while 23 (17 %) and eight (9 %) patients were treated for longer than 6 months by the PU and PNU, respectively. Treatment modalities adopted by pediatricians were significantly different from those preferred by pediatric nephrologists (Fig. 2c–e; Table 2).

### Symptomatic therapy

Symptomatic therapy at onset is shown in Table 3.

**Table 3:** Comparison of symptomatic therapy and hospitalization data at onset between the PUs and PNUs

Symptomatic therapy/hospitalization	Total patient cohort (*n* = 218)	Pediatrics units (*n* = 132)	Pediatric nephrology units (*n* = 86)	*p* value
Symptomatic therapy				
Albumin infusions	119 (54.5)	69 (52)	50 (58)	0.72
Diuretics	139 (63.7)	82 (62.1)	57 (66.2)	0.13
Vitamin D	95 (43.6)	51 (38.6)	44 (51.2)	0.068
Proton pump inhibitors or H2 antagonists	98 (44.9)	49 (22.5)	49 (57)	0.016
ASA	47 (21.5)	18 (13.6)	29 (33.7)	<0.0001
Calcium (carbonate or lactate)	14 (6.4)	9 (6.8)	5 (5.8)	0.34
Ca channel blockers, ACE inhibitors	9 (4.1)	4 (3)	5 (5.8)	0.30
Antibiotic prophylaxis	34 (15.6)	18 (13.6)	16 (18.6)	0.25
Hospitalization data				
Hospitalization (days)	10.7 (2–35)	10.9 (2–35)	10.4 (2–29)	0.41
Blood samples/days of hospitalization	0.5 (0.1–1.5)	0.49 (0.1–1.2)	0.47 (0.1–1.5)	0.70

Data are presented as the mean with the percentage in parenthesis, or as the median with the range in parenthesis

ASA, Acetylsalicylic acid; ACE angiotensin converting enzyme

#### Albumin infusions

In total, 119 (54.5 %) patients received albumin infusions (20 % solution), with no distinction seen between SS and steroid-resistant (SR) children, at a mean dose of 1 g/kg and a median number of infusions (range 1–16). Albumin infusions were associated with furosemide treatment in 94.1 % of cases. The overall percentage of infusions was similar for both the PUs (52 %) and PNUs (58 %), but the use of infusions varied considerably between individual centers, ranging from 8.4 to 87.5 % in centers with at least ten subjects enrolled in the study. Laboratory (serum albumin, proteins, creatinine, urea, electrolytes, PrU/CrU) and clinical data (BMI, sBP and dBP SDS, urine output) did not differ between infused and non-infused subjects (Table 4), even when PU and PNU behavior was compared. Patients who received albumin infusions showed a significantly higher risk of remission at a later date (11 vs. 8 days, respectively; OR 2.05, 95 % CI 1.11–3.79).

**Table 4:** Comparison of clinical and laboratory data of patients receiving or not receiving albumin infusions or thromboembolic prophylaxis^a^

Clinical and laboratory data						
Albumin infusion	Albumin (g/dL)	Serum Na (mmol/L)	Urine output (mL/kg/h)	sBP (SDS)	dBP (SDS)	BMI (SDS)
Yes	1.46 ± 0.4	135.3 ± 3.4	1.6 ± 1.1	1.2 ± 1.1	1.4 ± 1.0	0.9 ± 1.0
No	1.36 ± 0.4	137.1 ± 2.8	1.1 ± 0.8	0.9 ± 1.13	1.05 ± 0.8	0.6 ± 1.0
*p* value	NS	NS	NS	NS	NS	NS
Thromboembolic prophylaxis (ASA)	Albumin (g/dL)	Platelets (×10^3^/μL)	Antithrombin III (%)	Fibrinogen (mg/dL)	Cholesterol (mg/dL)	Triglycerides (mg/dL)
Yes	1.3 ± 0.4	438 ± 18.0	65 ± 4.0	687 ± 34	397 ± 12.2	196 ± 15.0
No	1.4 ± 0.4	430 ± 10.9	71 ± 4.1	651 ± 25	402 ± 8.5	217 ± 12.7
*p *value	NS	NS	NS	NS	NS	NS

sBP, Systolic blood pressure; dBP, diastolic blood pressure; NS, not significant

^a^This analysis did not reveal any difference between the two group in terms of Albumin infusion and thromboembolic prophylaxis. No differences were found when data were analyzed separately in children treated by pediatricians and pediatric nephrologists

#### Thromboembolic prophylaxis

Prophylactic therapy with acetylsalicylic acid was prescribed for 47 subjects (21.5 %). Hematological risk factors of thromboembolism (albumin, platelets, fibrinogen, antithrombin III, total cholesterol and triglycerides) did not differ between treated and non-treated subjects (Table 4), also when PU and PNU data were separately examined. Pediatric nephrologists prescribed thromboembolic prophylaxis more often than pediatricians (33.7 vs. 13.6 %, respectively; *p* < 0.0001).

#### Diuretics

Diuretic agents were used in 145/218 subjects (66.5 %). Furosemide was the most widely used diruretic agent, either alone (64.8 %) or in association with other diuretics (26.2 %) (Table 5). Diuretic treatment was not associated with a shorter time to remission. Urine output (1.6 ± 1.1 vs. 1.0 ± 0.7 mL/kg/h), serum albumin (1.37 ± 0.04 vs. 1.46 ± 0.06 g/dL), electrolytes, protein, urea and clinical parameters (weight-, BMI-, sBP- and dBP-SDS) did not differ between treated and non-treated subjects. Pediatricians used furosemide alone more frequently than pediatric nephrologists (45.4 vs. 39.5 %, respectively), while pediatric nephrologists used a combination of two diuretics more often than pediatricians (25.5 vs. 15.5 %, respectively).

**Table 5:** Diuretics utilized for the treatment of edema

Diuretic treatment	Results
No diuretics	73 (33.5)
Diuretics utilized	145 (66.5)
Furosemide	94 (64.8)
Furosemide + spironolactone	31 (21.4)
Furosemide + spironolactone + hydrochlorothiazide	5 (3.4)
Furosemide + hydrochlorothiazide	2 (1.4)
Spironolactone	6 (4.1)
Spironolactone + hydrochlorothiazide	3 (2.1)
Hydrochlorothiazide	3 (2.1)
Total diuretics (alone or in association)	
Furosemide	132 (91.0)
Spironolactone	46 (31.7)
Hydrochlorothiazide	13 (9.0)

Data are presented as the mean, with the percentage in parenthesis

#### Acute complications

Of the 218 pediatric patients, 27 (12.4 %) developed infections, with 16 children (7.3 %) having bacterial infections (8 pneumonia, 1 peritonitis, 1 cellulitis and 1 otitis; 5 were not specified), ten having viral infections (enteric or upper respiratory infections and 1 case of chickenpox), and one having a fungal infection. Thromboembolic complications were seen in two patients (pulmonary embolism and cerebral venous thrombosis; one patient had congenital dysfibrinogenemia).

## Discussion

Our epidemiological data (3.5 new cases/100,000 age-related population/year; male:female, 2:1) do not differ from those reported by previous retrospective [1, 2] or prospective [3, 24, 25] studies, confirming that INS is a rare disease. Various regional and national guidelines [12, 13, 26] have been developed, and the new Kidney Disease Improving Global Outcomes (KDIGO) guidelines have recently been published [14]. In Italy, shared treatment guidelines are lacking and, consequently, the choice of steroid regimen and symptomatic treatments still depends on the clinical expertise of each single unit. The aim of our study was thus to establish a shared protocol based on a retrospective evaluation of the different therapeutic strategies adopted.

### Steroid treatment

Our patients received a median PDN dose of 55.2 mg/m^2^/day. However, cases of either underdosing or overdosing (from 27 to 75 mg/m^2^) were noted. Comparison of PDN dosing in the PU and PNU revealed a significant difference (60 vs. 53.5 mg/m^2^, respectively). The general pediatricians in our study determined PDN doses using the body surface area (BSA)-based formula (60 mg/m^2^/day) for two-thirds of their patients, whereas they used the weight-based dosing formula (2 mg/kg/day) for fewer than one-third of their patients. In contrast, pediatric nephrologists utilized the weight-based formula more often (2/3 of patients). If we consider that the majority of children in the study cohort weighed <30 kg, the nephrologists’ preference for weight-based dosing could explain why their daily doses were lower than those of the general pediatricians, as mathematical models have demonstrated that weight-based dosing can be less than BSA-based dosing in smaller children [27].

Likewise, different steroid protocols were used. Overall, a 4-week induction therapy was the treatment of choice in one-half of the subjects, whereas a 6-week induction therapy was the treatment of choice in one-quarter of patients, while the remaining patients received either a much longer or much shorter induction therapy. At the time of our study, the Cochrane review suggesting the efficacy of longer treatment duration and higher cumulative steroid dose in reducing the risk of relapses had already been published [5], and the ISKDC protocol, recommending a cumulative dose of 2,240 mg/m^2^, was still widely used, especially by pediatricians. Concomitantly, extended protocols (APN protocol, total cumulative dose 3,360 mg/m^2^) [11] were gaining popularity among nephrologists. For this reason, the induction regimens in our study were longer (and cumulative steroid doses higher) in patients treated by nephrologists. We noted that pediatricians were using modified ISKDC regimens, with longer maintenance therapies, with progressive tapering to minimal PDN doses for up to 9 months, which explains why the total duration and cumulative PDN dose were significantly higher in the pediatricians’ group (24 weeks, 3,722 mg/m^2^) than in the pediatric nephrologists’ group (20 weeks, 3,301 mg/m^2^).

When the new 2012 KDIGO recommendations [14, 15] were applied to this cohort, in a retrospective simulation, non-adherence to the recommended range of PDN cumulative dose and/or length of steroid regimen was noted in 62/118 subjects (52.5 %), with more patients being “overtreated” than “undertreated” (37.2 vs. 15.2 %, respectively). The overall rate of non-adherence to KDIGO guidelines was significantly higher for patients treated by pediatricians (64.1 vs. 40.7 %, respectively; *p* = 0.01) than for those treated by pediatric nephrologists. This means that the group of undertreated patients from our cohort could have had a higher risk of relapse or steroid dependency, while the overtreated subjects would have been exposed to an increased risk of side-effects, probably without beneficial effects for the clinical course of the disease. More recently, a randomized controlled trial showed that PDN treatment (APN regimen) prolonged from 3 to 6 months without any increase in the cumulative dose did not benefit clinical outcome [28]. Applying this result to our cohort of patients, there would have been no advantage for children who took a cumulative dose equivalent to the APN protocol over a longer period of time.

Similar practice variations have been documented in two American and Canadian cohorts [29, 30]: in both studies, data were collected by means of a web-based survey completed by pediatric nephrologists. Striking variations were found in the number of daily steroid administrations (range 1–3) and in daily and alternate-day regimen duration. The authors of these studies expressed their hope of overcoming these discrepancies by creating a standardized clinical pathway to promote the uptake of the evidence published in the Guidelines into daily clinical practice. This is what has clearly come to light from the evaluation of our data—namely, guidelines do exist yet they are not adhered to in a correct way, leading to a great variation in the clinical management of INS.

### Symptomatic therapy

Noted differences were evident in our data. However, it must be acknowledged that guidelines are actually hard to develop for the symptomatic treatment of patients with NS due to the lack of trial data and the limitations of observational data.

#### Albumin infusions

Approximately one-half of our patients (54.5 %) received albumin infusions. To our knowledge, no epidemiological data have been published on the use of infusions in nephrotic patients at onset in large cohorts of patients. In nephrotic children, albumin may be essential when a circulatory volume depletion is present (underfilling hypothesis) [31, 32], while albumin infusion may be dangerous when the circulatory volume is not depleted (overfilling hypothesis) [33–36]. In the clinical setting, it is not easy to differentiate circulatory volume contraction from volume expansion, and various parameters, unfortunately not easily available in the routine clinical setting, have been proposed as biomarkers, such as the ratio urinary potassium (uK)/urinary sodium + uK (an index of aldosterone bioactivity) [37] or the inferior vena cava diameter [18]. Kapur et al. showed that a simple tool, the fractional excretion of sodium (FeNa), effectively differentiates hypo- from hypervolemic edematous children and that the use of diuretics without albumin is safe and beneficial in hypervolemic patients [16]. While these authors report that hypovolemia is present in about 30 % of nephrotic children, albumin was administered to 55 % of the children in our study. Unexpectedly, the serum chemistry and clinical data of the patients who received infusions did not differ from the data obtained from patients who were not treated with infusions. Significantly different practices regarding the use of infusions were noted between centers, confirming that the decision to administer albumin is often based on the single-center experience. Moreover, the patients who received albumin went into remission later than the untreated subjects (11 vs. 8 days, respectively). A similar result was described by Yoshimura et al. in a cohort of 27 adult patients with Ns, leading the authors to speculate that morphological changes in the glomerulus might be aggravated when albumin is administered [38]. Among our patients, 94 % were given albumin in association with intravenous furosemide; however, the efficacy of this combination therapy has been reported to be low and transient, and it remains controversial [19, 23, 36, 39–41].

#### Diuretics

In our study, two-thirds of the subjects were treated with diuretics, mainly (91 %) furosemide. Published data show that subjects with NS have an impaired natriuretic response to loop diuretics: low plasma albumin binding allows furosemide to leak out of the plasma into the interstitial space and delivery to the peritubular space is diminished [35, 42]. Thus, to achieve a good natriuretic response, it may be necessary to use high doses of furosemide (however there are no studies comparing different dosing levels) or add other diuretics to the treatment regimen [42, 43]. In our study, only 29 % of the patients were prescribed two diuretics. Despite frequent reports about its effectiveness, amiloride was never used [43, 44].

#### Thromboembolic prophylaxis

Only two cases of thromboembolism were reported among our patient cohort. While the incidence of thromboembolic complications in adults with NS [15, 45, 46] ranges from 10 to 42 %, that in children ranges from only 1.8 to 5.3 % and is more likely associated with congenital NS, membranous nephropathy or secondary NS [46–48]. For this reason, there is no consensus on the role of prophylactic anticoagulation [49]. Most clinicians do not initially prescribe prophylactic warfarin therapy [50]. Likewise, for high-risk patients, low-dose aspirin or dipyridamole are suggested by some guidelines [12] but not recommended by others [13], although no controlled trials have demonstrated their efficacy [17].

In our study, prophylactic therapy was performed in about 20 % of cases, exclusively with aspirin. Of note, laboratory levels of risk factors for thromboembolism did not differ between treated and non-treated subjects (Table 3). Prophylactic treatment was prescribed more frequently by pediatric nephrologists than by pediatricians (33.7 vs 13.6 %, respectively), possibly because pediatric nephrologists usually treat more complicated and severe forms of INS.

### Strengths and weakness of the study

The main strength of our study is the large number of participating centers and, more importantly, the number of non-specialized PUs involved, as most previous studies reported series of patients from tertiary units only. Further strengths include the rather short recruitment period and the evaluation of clinical and laboratory parameters rarely evaluated in previous studies. The major limitation of the study is its retrospective nature, especially in terms of the evaluation of clinical parameters and the number of patients lost to follow-up.

## Conclusions

This study has brought to light many inhomogeneities as regards the steroid treatment of INS children at onset and a lack of implementation of the current guidelines. Treatment and prevention of the acute complications significantly varies between centers and appears to be unrelated to any specific clinical or laboratory parameters. Therefore, shared guidelines for the symptomatic treatment and educational activities for the proper implementation of current guidelines on steroid treatment are necessary to avoid the above-mentioned unnecessary interventions.

A shared protocol for the treatment of the first episode of INS, involving both pediatricians and pediatric nephrologists, was implemented in the centers that took part in this study. To evaluate the impact of this common protocol (in terms of clinical results and socio-economic costs) an observational prospective study on the treatment of INS was officially initiated in 2011 (ClinicalTrials.gov Id.: NCT01386957).

## Appendix

**Members of the Nefrokid Study Group (all in Italy)**Andrea Pasini, Francesca Mencarelli, Chiara De Mutiis, Elena Monti, GiovanniMontini Nephrology and Dialysis Unit, Department of Pediatrics, Azienda OspedalieroUniversitaria Sant'Orsola-Malpighi, Bologna; Gabriella Aceto, Giovanni Messina, LauraSpagnoletta, Mario Giordano, Tommaso De Palo Nephrology Division, Giovanni XXIIIChildren's Hospital, Bari; Alberto Edefonti, Luciana Ghio, Gianluigi Ardissino, ElenaGroppali, Marta Lepore Pediatric Nephrology and Dialysis Unit, Fondazione Ca’ GrandaIRCCS Ospedale Maggiore Policlinico, Milan; Silvio Maringhini, Vitalba AzzolinaPediatric Nephrology Unit, Children's Hospital ‘G. Di Cristina’, A.R.N.A.S. ‘Civico’, Palermo;Carmelo Fede, Roberto Chimenz, Giovanni Conti Pediatric Nephrology and Dialysis,AOU G.Martino, Messina; Marco Materassi, Fiammetta Ravaglia Nephrology andDialysis Unit, Meyer Children's Hospital, Florence; Maria D’Agostino Pediatric Unit,S.Giovanni XXIII° Hospital,Bergam; Sante Cantatore Department of Pediatrics, AziendaOspedaliera-University of Modena, Modena; Anita Ammenti Department of Pediatrics,University of Parma, Parma; Chiara Gualeni Pediatric Unit, Children’s Hospital, Brescia;Elena Cama Department of Pediatrics and Neonatology, Desenzano del Garda; MariottiPaola Pediatric Unit, San Jacopo Hospital, Pistoia; Amata Negri Pediatric Unit , FilippoDel Ponte Hospital, Varese; Alberto Bettinelli Pediatric Unit, San Leopoldo MandicHospital, Merate; Alessandra Lavacchini Pediatric Unit, Ospedale degli Infermi, Rimini;Alessandra Dozza Pediatric Unit, Ospedale Maggiore, Bologna; Angela Simoni PediatricUnit, Ramazzini Hospital, Carpi; Marina Piepoli Pediatric Unit, Guglielmo da SalicetoHospital, Piacenza, Italy; Felice Sica Pediatric Unit, AOU Ospedali Riuniti, Foggia;Gabriele Ripanti Pediatric Unit, San Salvatore Hospital, Pesaro; Marina Milani PediatricUnit, Fondazione MBBM, S. Gerardo Hospital, Monza; Paola Tommasi Pediatric Unit,Vittore Buzzi Hospital, Milan; Carla Romanello Pediatric Unit, S. Maria della MisericordiaHospital, Udine; Manuela Pasini Pediatric Unit, Maurizio Bufalini Hospital, Cesena; PaolaMastinu Pediatric Unit, S.Chiara Hospital, Trento; Laura Luti Pediatric Unit, AOU Pisa;Antonella Amendolea Pediatric Unit, Cecina; Silvia Manfredi Pediatric Unit, Massa eCarrara; Emanuela Lanfranchi Pediatric Unit, Fermo; Maria Principi Pediatric Unit,Macerata; Agrippino Reciputo Pediatric Unit, Cinisello Balsamo; Marialuisa CascianaPediatric Unit, C. Poma Hospital, Mantova; Antonio Pellegatta Pediatric Unit, BustoArsizio; Fiorella Russo Pediatric Unit, Desio; Nicola Altamura Pediatric Unit, Sesto SanGiovanni; Lorena Ruzza Pediatric Unit, San Carlo Hospital, Milan; Stefano SardiniPediatric Unit, Asola; Ines L'Erario Pediatric Unit, Burlo Garofalo Hospital, Trieste;Claudio Ruberto, Antonella Crisafi Pediatric Unit, Santa Maria Nuova Hospital, ReggioEmilia; Andrea Zucchini Pediatric Unit, Faenza; Laura Serra Pediatric Unit, Santa Mariadella Scaletta Hospital, Imola; Peter Bertamini Pediatric Unit, Santa Maria del CarmineHospital, Rovereto; Rita Bini Pediatric Unit, Grosseto; Patrizia Cortesi Pediatric Unit,Pescia; Caterina Balducci Pediatric Unit, Prato; Francesca Simoni Pediatric Unit, Pianadi Lucca Hospital, Lucca; Patrizia Fonduli, Franca Paola Zurrida Pediatric Unit, BrotzuHospital, Cagliari; Laura De Petris Pediatric Unit, Mazzoni Hospital, Ascoli Piceno;VinicioGoj Pediatric Unit, Fatebenefratelli Hospital, Milan; Gian Luigi Marsiglia Pediatric Unit,IRCCS Policlinico S Matteo, Pavia; Patrizia Caruso Pediatric Unit, Cremona; FilippoSalviniPediatric Unit, S. Paolo Hospital, Milano; Paola Perotti Pediatric Unit, Voghera;Anna Bussolini Pediatric Unit, Tradate; Stefano Poli, Barbara Balduzzi Pediatric Unit,Esine; Barbara Roman Pediatric Unit, Vimercate; Sergio Mariani, Laura CafarelliPediatric Unit, Saronno; Vittorio Venturoli Pediatric Unit, Morgagni-Pierantoni Hospital,Forlì; Andrea Corsini Pediatric Unit, Bentivoglio; Luca Casadio, Fabrizio PugliesePediatric Unit, Ravenna.
